# From Prevalence to Prediction: A Cross-Sectional Study to Develop a Novel Risk Score for Hearing Loss in High-Risk Neonates in South India

**DOI:** 10.7759/cureus.118020

**Published:** 2026-10-16

**Authors:** Sangeetha T Sachithanandam, Rajeswari Baskaradass

**Affiliations:** 1 Otolaryngology - Head and Neck Surgery, Employees’ State Insurance Corporation (ESIC) Medical College and Hospital, Chennai, IND; 2 Otolaryngology - Head and Neck Surgery, JR Medical College and Hospital, Dindivanam, IND

**Keywords:** hearing loss, high-risk neonates, predictive factors, prevalence, receiver operating characteristic curve

## Abstract

Background and aim: Neonates admitted to the neonatal intensive care unit (NICU) with multiple risk factors are more likely to develop hearing loss. This study aimed to determine the prevalence of hearing loss, identify its predictive factors, and develop a novel clinical risk score for hearing loss among high-risk neonates.

Materials and methods: This cross-sectional study included 105 high-risk neonates admitted to the NICU from July 2024 to June 2025. The hearing assessment protocol included transient-evoked otoacoustic emissions at discharge and at six weeks of corrected age, followed by diagnostic brainstem-evoked response audiometry at three months. Associations between risk factors and hearing loss were analyzed using logistic regression. A novel clinical risk score was developed using the factors significantly associated with hearing loss.

Results: The prevalence of hearing loss among high-risk neonates was 4.8% (95% CI, 1.6%-10.8%). Hearing loss was significantly associated with intrauterine toxoplasmosis, perinatal asphyxia, neonatal hyperbilirubinemia (NNH) requiring exchange transfusion, stage II or higher hypoxic-ischemic encephalopathy, neonatal sepsis, neonatal seizures, syndromic or craniofacial anomalies, and ototoxic drug exposure (p < 0.05). The clinical risk prediction model, developed using significant predictive factors and assigning double weight to intrauterine toxoplasmosis and NNH requiring exchange transfusion, achieved a sensitivity of 60% and a specificity of 96% at a cutoff score of ≥3.

Conclusions: The prevalence of hearing loss among high-risk neonates was 4.8%. The novel clinical risk score developed in this study may help guide targeted screening, timely diagnosis, and early intervention for hearing loss.

## Introduction

Auditory stimulation during the prelingual period is essential for normal speech and language development in children. Undiagnosed and untreated hearing loss during the first three years of life has been associated with significant deficits in speech, language, and cognitive development, which can subsequently lead to poor educational outcomes and impaired emotional, psychosocial, and vocational development. Studies have shown that enrolling children with hearing loss in early intervention programs before 12 months of age, combined with high levels of family involvement, results in significantly better language development and reasoning skills at five years of age than enrolling them later [1,2]. Children whose hearing loss was identified and addressed before six months of age also demonstrated significantly higher language development scores than those whose hearing loss was identified and addressed later [3].

According to the World Health Organization, an estimated 7.5 million children younger than five years worldwide had hearing impairment. In India, the National Sample Survey (2002) identified hearing impairment as the second most common cause of disability among children [4]. Studies have reported hearing loss incidence rates of 1.59-8.8 per 1,000 live births among well neonates and 7-49 per 1,000 live births among high-risk neonates in India [5]. Since the implementation of Universal Newborn Hearing Screening in India in 2013, several studies have demonstrated the effectiveness of otoacoustic emissions (OAE) and brainstem-evoked response audiometry (BERA) in improving the early identification of newborn hearing loss and facilitating timely intervention [6]. Although studies have documented various perinatal risk factors for hearing loss in neonates, a clinical risk score for neonates with multiple coexisting risk factors is lacking.

The primary aim of this study was to assess the prevalence of hearing loss and identify its predictive factors in high-risk neonates. The secondary objective was to develop a novel clinical risk score for hearing loss in neonates with multiple coexisting risk factors.

## Materials and methods

This cross-sectional study was conducted at Employees’ State Insurance Corporation (ESIC) Medical College and Hospital, a tertiary care institute in Chennai, India, from July 2024 to June 2025. The Institutional Ethics Committee of ESIC Medical College and Hospital granted approval (IEC/2025/01/28). A waiver of informed consent was obtained.

Term and preterm neonates admitted to the neonatal intensive care unit (NICU) were enrolled if they were classified as high-risk based on the presence of one or more risk factors specified in the Joint Committee on Infant Hearing (JCIH) guidelines [7,8]. The inclusion criteria were preterm birth at less than 37 weeks of gestation; perinatal asphyxia, defined as an Apgar score of less than seven out of 10 at five minutes [8]; very low birth weight (<1,500 g); meconium-stained amniotic fluid; respiratory distress syndrome (RDS) requiring assisted ventilation for more than two days; NICU stay exceeding five days; neonatal sepsis; neonatal seizures; meningitis (bacterial or viral, including herpes and varicella); neonatal hyperbilirubinemia (NNH) requiring phototherapy or exchange transfusion; exposure to ototoxic drugs; craniofacial anomalies; Rh incompatibility; metabolic disorders (hypothyroidism, hyperthyroidism, congenital adrenal hyperplasia, galactosemia, or phenylketonuria); syndromic associations (neurofibromatosis, osteopetrosis, Usher syndrome, Waardenburg syndrome, Pendred syndrome, Alport syndrome, or Jervell and Lange-Nielsen syndrome); intrauterine infections (toxoplasmosis, rubella, cytomegalovirus, histoplasmosis, human immunodeficiency virus (HIV), syphilis, measles, or varicella); and intrauterine growth restriction (IUGR). Neonates whose parents did not consent to BERA were excluded. Data were collected from NICU records, the high-risk infant register, and the newborn hearing screening register maintained by the Department of Audiology and Speech-Language Pathology using a prespecified data collection form.

High-risk neonates underwent a two-stage hearing screening protocol. The first stage involved a clinical examination of both ears to identify debris, skin tags, auricular or external auditory canal deformities, and ear infections. Transient-evoked otoacoustic emissions (TEOAEs), which assess cochlear outer hair cell function, were then measured before NICU discharge using the MAICO ERO•SCAN screener (MAICO Diagnostics GmbH, Berlin, Germany). A click stimulus was presented at an intensity of 83 dB sound pressure level, and responses were analyzed across six frequency bands centered at 1.5, 2.0, 2.5, 3.0, 3.5, and 4.0 kHz. The default screening protocol used a maximum averaging time of 64 seconds, with a passing criterion of a signal-to-noise ratio of at least 4 dB in three or more of the six frequency bands. The instrument automatically terminated testing when the passing criteria were met or the maximum test duration was reached. Results were recorded as “Pass” or “Refer” for each ear separately.

Repeat TEOAE screening was performed at six weeks of age for term neonates, in conjunction with routine vaccination. For preterm neonates, screening was scheduled according to corrected age. All neonates subsequently underwent detailed BERA testing at three months of age to assess the integrity of the auditory pathway. Auditory brainstem responses were recorded using the GSI Audera auditory evoked potential system (Grason-Stadler). Click stimuli were delivered through insert earphones using rarefaction polarity at intensities ranging from 90 to 30 dB normalized hearing level (nHL). The stimulus repetition rate was 11.1 clicks per second, and 1,500 responses were averaged for each recording. The recorded responses were filtered using a band-pass filter of 30-1,500 Hz. The electrode montage consisted of an active electrode on the mastoid of the test ear, a reference electrode on the forehead, and a ground electrode on the mastoid of the nontest ear. Recordings were obtained at different stimulus intensities, with two replications at each intensity to ensure waveform reproducibility. The absolute latencies of waves I, III, and V and the interpeak latencies I-III, III-V, and I-V were assessed whenever the waves were identifiable.

Normal hearing was defined as an electrophysiological hearing threshold of ≤30 dB nHL, with absolute and interpeak latencies within the expected limits for gestational age and wave responses consistent with established BERA criteria. At three months of age, hearing loss in high-risk neonates was classified according to Goodman’s classification as follows: 0-15 dB, normal; 16-25 dB, minimal; 26-40 dB, mild; 41-55 dB, moderate; 56-70 dB, moderately severe; 71-90 dB, severe; and greater than 90 dB, profound. Auditory dyssynchrony was also assessed by combining OAE and BERA findings. Sex distribution and the associations between hearing loss and the type and number of risk factors were also analyzed.

The sample size was calculated assuming a 95% confidence level, an alpha error of 5%, an estimated prevalence of 13.3%, and an absolute error of 6.65% [9]. Based on these assumptions, the required sample size was 100 neonates. Consecutive eligible neonates were enrolled in the study.

Statistical analysis

Data were entered into Excel (Microsoft Corp., Redmond, WA, USA) and imported into STATA version 12 (StataCorp LP, College Station, TX, USA) for analysis. Baseline characteristics were summarized using means and standard deviations (SDs) or medians and interquartile ranges (IQRs) for continuous variables, as appropriate, and frequencies and percentages for categorical variables. Penalized logistic regression was performed to assess the associations between BERA-determined hearing loss and clinical risk factors. Results were reported as crude odds ratios (ORs), 95% confidence intervals (CIs), and p-values. Statistical significance was defined as p < 0.05.

Based on the logistic regression results, a novel clinical risk score for hearing loss was developed using clinical variables available during the neonatal period. A receiver operating characteristic (ROC) curve was constructed to determine a clinically acceptable cutoff, and sensitivity and specificity were reported at different cutoff points. The area under the ROC curve (AUC) was also calculated.

## Results

The flow of high-risk neonates through the study is presented in Figure 1. Of the 1,124 live births during the study period, 384 neonates were admitted to the NICU, of whom 120 met the eligibility criteria for high-risk neonates. Fifteen neonates were lost to follow-up after discharge. Thus, 105 high-risk neonates were included in the final analysis, of whom 47.6% (n = 50) were male.

**Figure 1:**
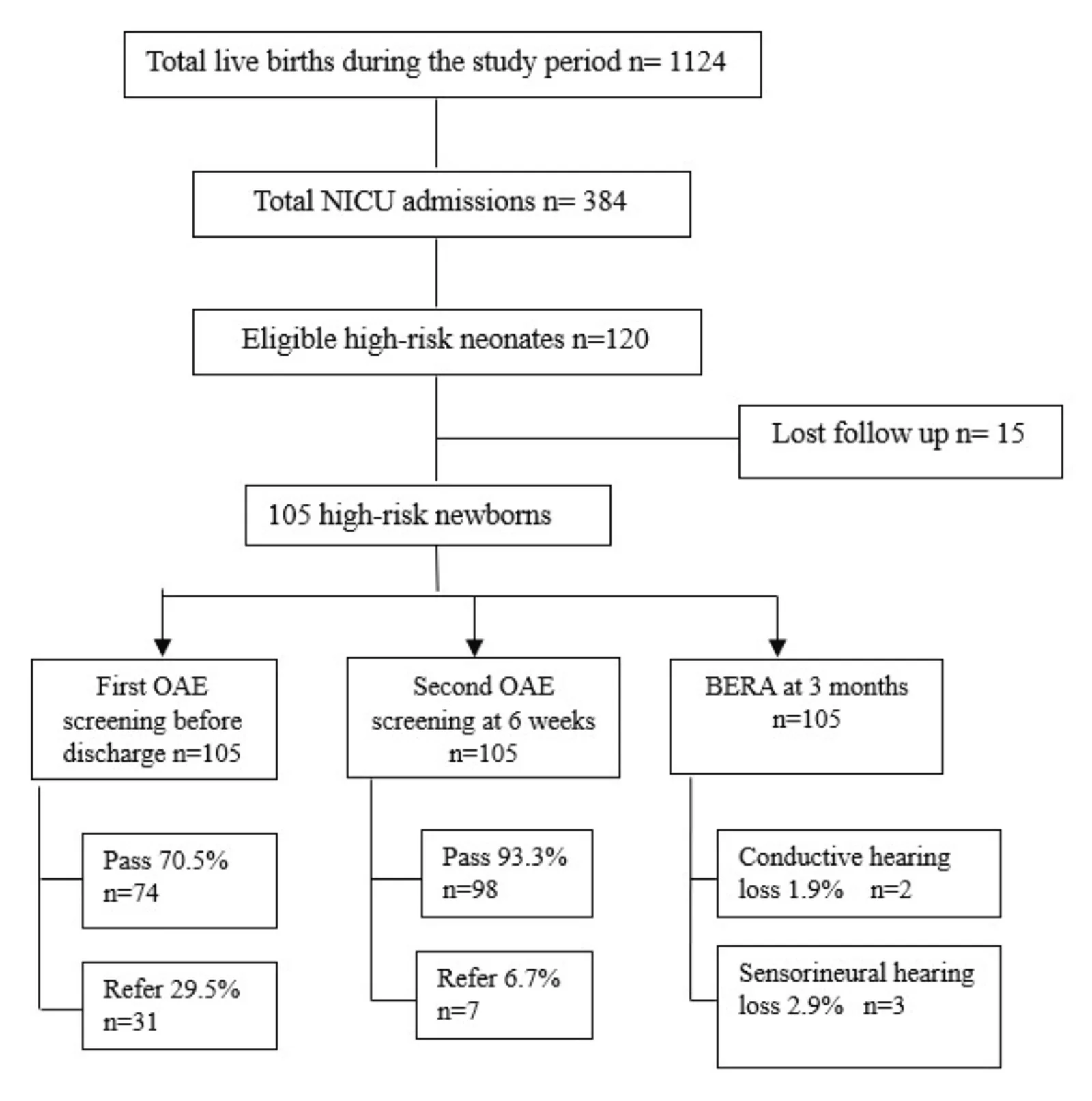
Study flow diagram illustrating the screening test results of high-risk neonates NICU: neonatal intensive care unit, OAE: otoacoustic emissions

The mean birth weight was 1,760 g (SD, 570 g), and the median was 1,700 g (IQR, 1,400-2,100 g). The mean gestational age was 33 weeks and six days (SD, four weeks and two days), and the median was 33 weeks (IQR, 32-36 weeks).

All 105 high-risk neonates underwent initial TEOAE screening before NICU discharge. Of these, 70.5% (n = 74) passed the screening, whereas 29.5% (n = 31) received a "Refer" result. The neonates underwent repeat TEOAE screening at six weeks of corrected age. Of the 105 neonates who underwent the second screening, 93.3% (n = 98) passed, whereas 6.7% (n = 7) did not pass the test.

Among the 105 neonates who underwent BERA, five were diagnosed with hearing loss. The prevalence of hearing loss among high-risk neonates was 4.8% (95% CI, 1.6-10.8%). Table 1 presents the percentage distribution of prenatal, natal, and postnatal risk factors among high-risk neonates, along with their OAE and BERA results.

**Table 1:** Distribution of prenatal, natal, and postnatal risk factors among high-risk neonates and their hearing test results OAE: otoacoustic emissions, BERA: brainstem-evoked response audiometry, NNH: neonatal hyperbilirubinemia, RDS: respiratory distress syndrome, HIE: hypoxic-ischemic encephalopathy

Risk factor	Present (n = 105)	OAE "refer" result (n = 31)	BERA hearing loss (n = 5)
Prenatal risk factors			
Rh incompatibility	6 (6%)	2 (6%)	-
TORCH infections - toxoplasmosis	1 (1%)	1 (3%)	1 (20%)
Natal risk factors			
Very low birth weight (1,000-1,500 g)	32 (30%)	16 (52%)	3 (60%)
Extremely low birth weight (<1,000 g)	7 (7%)	6 (19%)	-
Very preterm 28-32 weeks	44 (42%)	13 (42%)	1 (20%)
Extremely preterm less than 28 weeks	7 (7%)	6 (19%)	1 (20%)
Postnatal risk factors			
NNH - required phototherapy	28 (27%)	15 (48%)	1 (20%)
NNH - required exchange transfusion	1 (1%)	1 (3%)	1 (20%)
Perinatal asphyxia	14 (13%)	14 (45%)	3 (60%)
Mechanical ventilation - 2 days or more	1 (1%)	1 (3%)	1 (20%)
Intrauterine growth restriction	23 (22%)	12 (39%)	2 (40%)
HIE (stage II or higher)	4 (4%)	4 (13%)	1 (20%)
RDS	32 (30%)	16 (52%)	1 (20%)
Sepsis - viral/bacterial infections	11 (10%)	11 (35%)	2 (40%)
Neonatal seizures	4 (4%)	3 (10%)	1 (20%)
Syndromic hearing loss - Goldenhar syndrome	1 (1%)	1 (3%)	1 (20%)
Craniofacial anomalies - microtia	1 (1%)	1 (3%)	1 (20%)
Craniofacial anomalies - microcephaly	1 (1%)	-	-
Craniofacial anomalies - macrocephaly	1 (1%)	-	-
Neural tube defect	2 (2%)	1 (3%)	-
Intrahepatic biliary atresia	1 (1%)	-	-
Parasitic rachiphagus - conjoined twins	1 (1%)	-	-
Congenital heart disease	12 (11%)	8 (26%)	-
Exposure to ototoxic drugs	11 (10%)	11 (35%)	2 (40%)

Table 1 summarizes the distribution of prenatal, natal, and postnatal risk factors among the 105 high-risk neonates. The most common risk factors were prematurity (48%), RDS (30%), very low birth weight (30%), NNH requiring phototherapy (27%), and IUGR (22%). Among the five neonates diagnosed with hearing loss by BERA, perinatal asphyxia and very low birth weight were each observed in three cases (60%). Neonatal sepsis and exposure to ototoxic drugs were each present in two cases (40%). Intrauterine toxoplasmosis, NNH requiring exchange transfusion, and several neurological and craniofacial risk factors were also observed among neonates with hearing loss.

Of the five neonates diagnosed with hearing loss, two had unilateral moderate conductive hearing loss, representing 1.9% of the study population. Both were born at term and were appropriate for gestational age. One had unilateral microtia with external auditory meatal atresia, while the other had Goldenhar syndrome with external auditory meatal and canal atresia.

The remaining three neonates (2.9% of the study population) had bilateral sensorineural hearing loss, with severity ranging from mild to profound. The first neonate had mild sensorineural hearing loss in the right ear and moderate sensorineural hearing loss in the left ear. The neonate was born at 32 weeks of gestation, weighed 1,300 g at birth, and had perinatal asphyxia and NNH requiring exchange transfusion. The second neonate had profound sensorineural hearing loss in the right ear and mild sensorineural hearing loss in the left ear. The neonate was born at 28 weeks of gestation, weighed 1,000 g at birth, and had IUGR, perinatal asphyxia, stage II hypoxic-ischemic encephalopathy (HIE), and seizures. The neonate also required mechanical ventilation for five days because of RDS, had neonatal sepsis, and was exposed to ototoxic drugs. The third neonate had bilateral moderate sensorineural hearing loss and weighed 1,500 g at birth. The associated risk factors included perinatal asphyxia, intrauterine toxoplasmosis, NNH requiring phototherapy, sepsis, and exposure to ototoxic drugs. None of the neonates who passed TEOAE screening were diagnosed with hearing loss by BERA, and no cases of auditory dyssynchrony were identified.

The associations between risk factors and hearing loss are presented in Table 2. The study found statistically significant associations (p < 0.05) between hearing loss and intrauterine toxoplasmosis, perinatal asphyxia, NNH requiring exchange transfusion, stage II or higher HIE, neonatal sepsis, neonatal seizures, syndromic or craniofacial anomalies, and exposure to ototoxic drugs.

**Table 2:** Association of risk factors with hearing loss OR: odds ratio, CI: confidence interval, NNH: neonatal hyperbilirubinemia, RDS: respiratory distress syndrome, HIE: hypoxic-ischemic encephalopathy

Variable	Unadjusted penalized OR (95% CI)	p-value
Prenatal risk factors		
Rh incompatibility	1.32 (0.07-26.6)	0.86
Intrauterine toxoplasmosis	67.00 (2.38-1885.07)	0.01
Natal risk factors		
Birth weight <1.5 kg	2.37 (0.45-12.62)	0.31
Gestational age >32 weeks	1.000	Reference
Very preterm 28-32 weeks	0.51 (0.07-3.58)	0.50
Extremely preterm less than 28 weeks	3.40 (0.42-27.15)	0.25
Intrauterine growth restriction	2.64 (0.49-14.34)	0.26
Perinatal asphyxia	10.89 (1.92-61.78)	0.01
NNH	1.91 (0.36-10.26)	0.45
NNH with exchange transfusion	67.00 (2.38-1885.07)	0.01
HIE - stage 2	9.29 (1.10-78.41)	0.04
RDS	0.74 (0.11-4.89)	0.75
Postnatal risk factors		
Sepsis - viral/bacterial infections	6.88 (1.19-39.79)	0.03
Neonatal seizures	9.29 (1.10-78.41)	0.04
Syndromic or craniofacial anomalies	19.90 (2.85-138.73)	0.003
Ototoxic drugs	6.88 (1.19-39.79)	0.03

A novel clinical risk score was developed using the study findings to predict hearing loss, as determined by BERA, at three months of age. The scoring system incorporated clinical variables that could be assessed during the neonatal period and demonstrated an AUC of 0.95 (95% CI, 0.91-1.00) (Figure 2).

**Figure 2:**
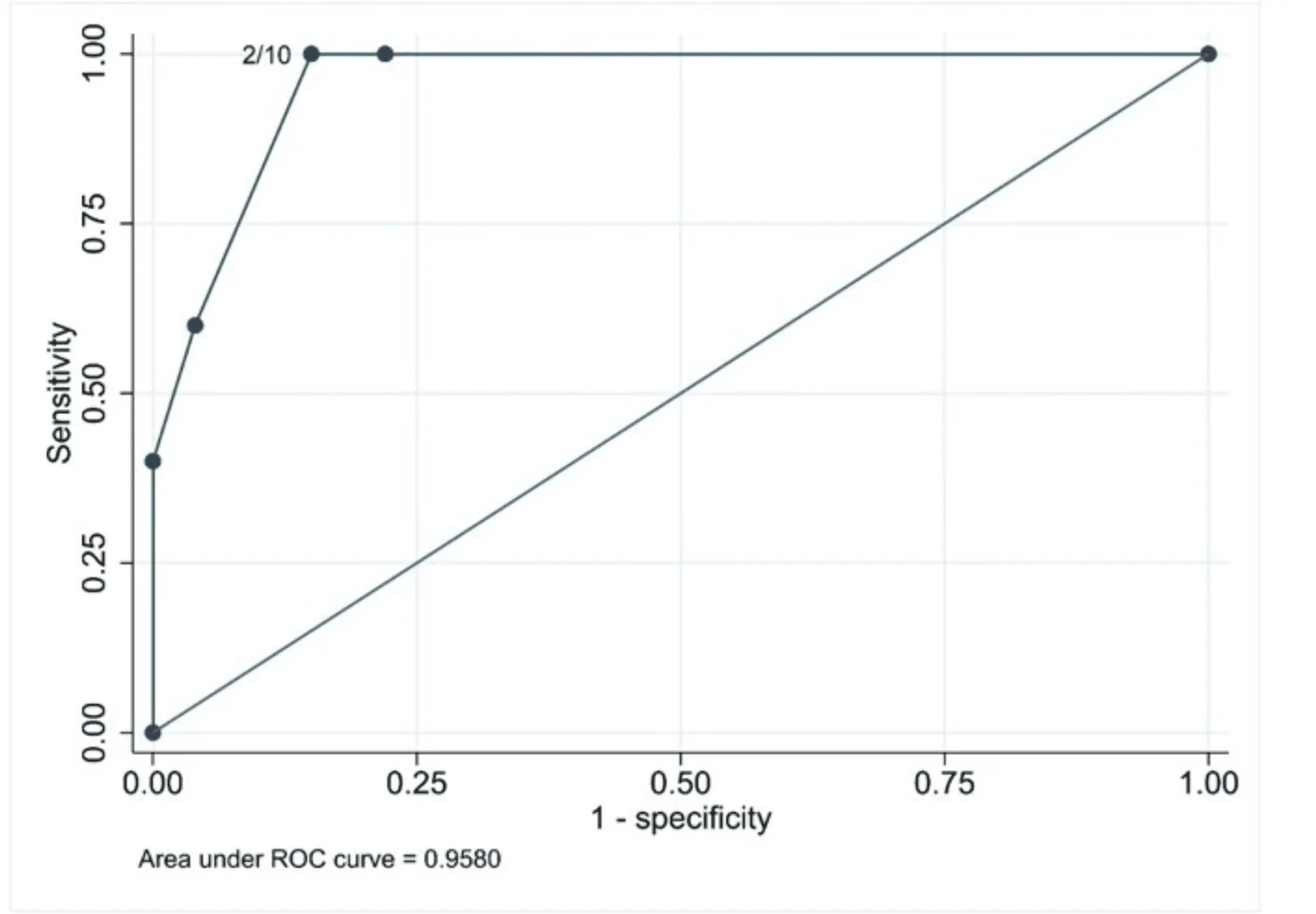
ROC curve used to determine the clinically acceptable cutoff and assess sensitivity and specificity at different cutoff points ROC: receiver operating characteristic

The clinical risk score comprised prenatal toxoplasmosis, perinatal asphyxia, NNH requiring exchange transfusion, HIE (stage II or higher), neonatal sepsis, neonatal seizures, craniofacial anomalies, and exposure to ototoxic drugs (Table 3). Double weight was assigned to prenatal toxoplasmosis and NNH requiring exchange transfusion. The clinical risk prediction model achieved a sensitivity of 100% and specificity of 78% at a cutoff score of 1, a sensitivity of 100% and specificity of 85% at a cutoff score of 2, a sensitivity of 60% and specificity of 96% at a cutoff score of ≥3, and a sensitivity of 40% and specificity of 100% at a cutoff score of ≥4.

**Table 3:** Predictive score components NNH: neonatal hyperbilirubinemia, HIE: hypoxic-ischemic encephalopathy

Risk factors	Score
Intrauterine toxoplasmosis	2
Perinatal asphyxia	1
NNH requiring exchange transfusion	2
HIE (stage II or higher)	1
Neonatal sepsis	1
Neonatal seizures	1
Craniofacial anomaly	1
Exposure to ototoxic drugs	1
Maximum possible score	10

## Discussion

This study found a 4.8% prevalence of hearing loss among high-risk neonates (95% CI, 1.6-10.8%). Hearing loss was significantly associated with prenatal toxoplasmosis infection, perinatal asphyxia, NNH requiring exchange transfusion, stage II or higher HIE, neonatal sepsis, neonatal seizures, syndromic or craniofacial anomalies, and exposure to ototoxic drugs (p < 0.05). A novel clinical risk score was developed to predict hearing loss, with double weight assigned to prenatal toxoplasmosis and NNH requiring exchange transfusion. At a cutoff score of ≥3, the model achieved a sensitivity of 60% and a specificity of 96%.

The JCIH recommends universal hearing screening by one month of age, diagnostic audiological evaluation by three months of age for infants who do not pass screening, enrollment in early intervention services by six months of age, and a family-centered approach [7]. Neonates admitted to the NICU, particularly those with multiple risk factors, require close monitoring and follow-up for hearing loss [8]. The present study enrolled 105 eligible high-risk neonates during the study period, of whom 47.6% were male and 52.4% were female. A similar study screened 100 high-risk neonates, of whom 59% were male and 41% were female [10].

In India, a two-stage sequential hearing screening protocol involving OAE and BERA is currently used under the Universal Newborn Hearing Screening Program. Both tests have been reported to facilitate the early identification of hearing loss before six months of age [11,12]. OAE assesses cochlear outer hair cell function and is preferred for initial newborn screening because it is simple, relatively quick, and cost-effective [13]. The second OAE screening following the initial assessment has been reported to have a sensitivity of 46.15%, specificity of 85.96%, positive predictive value of 42.85%, and negative predictive value of 87.5% [14]. BERA assesses auditory nerve function and evaluates the auditory pathway from the cochlea to the brainstem. Diagnostic BERA following OAE screening is essential for confirming hearing loss and identifying auditory neuropathy spectrum disorders in high-risk infants [7,12,14]. In the present study, all 105 high-risk neonates underwent initial TEOAE screening before NICU discharge. Of these, 29.5% failed the initial screening, and 6.7% failed the second OAE screening. In a study by Hardani et al., 530 NICU neonates were screened; 78 (11%) did not pass the initial OAE screening, and 28 (5.28%) did not pass the second screening [15].

Five neonates were diagnosed with hearing loss by diagnostic BERA in our study. Two had unilateral moderate conductive hearing loss (1.9%), while three had bilateral sensorineural hearing loss of varying degrees (2.9%). In a 2020 study conducted in India, Shukla et al. screened 100 neonates and identified hearing loss in two infants by BERA; both had bilateral sensorineural hearing loss of varying degrees [10]. Van Riper et al. also reported that bilateral hearing loss was predominant among high-risk neonates [16].

In the present study, the prevalence of hearing loss among high-risk neonates was 4.8%. Previous research has reported that the prevalence of hearing loss among high-risk NICU neonates is 6.5 times that among well neonates [17]. Vashistha et al. reported a prevalence of 15% among high-risk neonates in India in 2015 [18], while Gulati et al. reported a substantially higher prevalence of 18.57% among preterm infants in India in 2020 [14]. Other studies have reported prevalence rates ranging from 2% to 11% among high-risk neonates [15,18-20].

We observed a significant association between intrauterine toxoplasmosis and hearing loss. Rh incompatibility was not significantly associated with hearing loss, and none of the neonates had a family history of childhood hearing loss. Andrade et al. reported congenital toxoplasmosis as a significant risk factor for hearing loss in newborns [21,22].

Neither prematurity nor very low birth weight was significantly associated with hearing loss in the present study. In contrast, previous studies have reported significant associations between these factors and hearing loss [14,18].

Other factors significantly associated with hearing loss in our study included perinatal asphyxia, NNH requiring exchange transfusion, stage II or higher HIE, neonatal sepsis, neonatal seizures, exposure to ototoxic drugs, and syndromic or craniofacial anomalies. RDS and IUGR were not significantly associated with hearing loss. Poonual et al. identified craniofacial anomalies, low Apgar scores, sepsis, and exposure to ototoxic drugs as significant risk factors for hearing loss in infants [23]. Gouri et al. reported that severe birth asphyxia accompanied by fetal distress and low Apgar scores was associated with hearing loss in newborns [24]. Other studies have documented significant associations between hearing loss and birth asphyxia and NNH requiring exchange transfusion [10,14,18,25]. Van Riper et al. reported that craniofacial anomalies accounted for the largest proportion of hearing loss diagnoses among high-risk neonates [16]. Meyer et al. identified meningitis, sepsis, craniofacial anomalies, and a family history of hearing loss as significant risk factors [26]. However, none of the neonates in the present study had meningitis or a family history of hearing loss.

A novel clinical risk score was developed based on the study findings to predict hearing loss, with double weight assigned to intrauterine toxoplasmosis and NNH requiring exchange transfusion. The score comprised prenatal toxoplasmosis, perinatal asphyxia, NNH requiring exchange transfusion, stage II or higher HIE, neonatal sepsis, neonatal seizures, craniofacial anomalies, and exposure to ototoxic drugs. The scoring system demonstrated an AUC of 0.95 (95% CI, 0.91-1.00). The model achieved a sensitivity of 100% and specificity of 78% at a cutoff score of 1, a sensitivity of 100% and specificity of 85% at a cutoff score of 2, a sensitivity of 60% and specificity of 96% at a cutoff score of ≥3, and a sensitivity of 40% and specificity of 100% at a cutoff score of ≥4.

The ROC curve and AUC summarize the model's ability to discriminate between neonates with and without hearing loss. The ROC curve illustrates the trade-off between sensitivity and the false-positive rate across different thresholds, while the AUC quantifies overall discriminatory performance. An AUC closer to 1.0 indicates better discrimination, whereas an AUC of 0.5 indicates performance equivalent to chance. Although an AUC above 0.8 is generally considered indicative of good discrimination, the clinical utility of a prediction model also depends on the trade-off between missed diagnoses and unnecessary interventions. CIs around the AUC provide information about the precision of the estimate and should be considered when comparing models. These findings are consistent with the use of ROC-AUC analysis to evaluate the discriminatory performance of diagnostic and screening tools [27].

A strength of this study is the development of a novel clinical risk score that may serve as an assessment tool for high-risk neonates with multiple coexisting risk factors. The model may help identify neonates who require targeted screening, early diagnosis, and timely intervention for hearing loss.

A limitation of this study is the absence of external validation. Future prospective, multicenter validation studies are needed to assess the model's predictive accuracy and generalizability across diverse populations before it can be integrated into routine newborn hearing screening programs.

## Conclusions

This study highlights the importance of comprehensive hearing assessment and risk-based follow-up among high-risk neonates. The findings underscore the clinical relevance of identifying specific prenatal, perinatal, and postnatal risk factors to help prioritize hearing surveillance in this vulnerable population.

The novel clinical risk score developed in this study may serve as a tool for stratifying high-risk neonates according to their risk of hearing loss and guiding targeted audiological assessment. By helping identify infants who require closer monitoring, this approach could facilitate timely diagnosis and early intervention, potentially improving developmental outcomes. However, the score should be considered preliminary. Larger, multicenter, prospective studies are needed to validate its predictive performance and establish its applicability in routine clinical practice.

## References

[1] Moeller MP (2000). Early intervention and language development in children who are deaf and hard of hearing. Pediatrics.

[2] Bubbico L, Di Castelbianco FB, Tangucci M, Salvinelli F (2007). Early hearing detection and intervention in children with prelingual deafness, effects on language development. Minerva Pediatr.

[3] Yoshinaga-Itano C, Sedey AL, Coulter DK, Mehl AL (1998). Language of early- and later-identified children with hearing loss. Pediatrics.

[4] Ansari MS, Sood AS, Gill JS (2022). National infant screening for hearing program in india: necessity, significance and justification. Indian J Otolaryngol Head Neck Surg.

[5] Rawat V, Arora R, Singh J, Gupta A (2023). Incidence of hearing loss in neonates at a secondary care hospital in North India-a pilot UNHS study. Egypt J Otolaryngol.

[6] Upadhyay K, Gupta V, Singh S (2022). Outcome of universal neonatal hearing screening programme at a tertiary care centre: a prospective study. Indian J Otolaryngol Head Neck Surg.

[7] (2007). Year 2007 position statement: principles and guidelines for early hearing detection and intervention programs. Pediatrics.

[8] Hajare P, Mudhol R (2022). A study of jcih (joint commission on infant hearing) risk factors for hearing loss in babies of nicu and well baby nursery at a tertiary care center. Indian J Otolaryngol Head Neck Surg.

[9] Colella-Santos MF, Hein TA, de Souza GL, do Amaral MI, Casali RL (2014). Newborn hearing screening and early diagnostic in the NICU. Biomed Res Int.

[10] Shukla A, Hosamani P (2022). Role of hearing screening in high-risk newborns. Indian J Otolaryngol Head Neck Surg.

[11] Escobar-Ipuz FA, Soria-Bretones C, García-Jiménez MA, Cueto EM, Torres Aranda AM, Sotos JM (2019). Early detection of neonatal hearing loss by otoacoustic emissions and auditory brainstem response over 10 years of experience. Int J Pediatr Otorhinolaryngol.

[12] Kirkim G, Serbetcioglu B, Erdag TK, Ceryan K (2008). The frequency of auditory neuropathy detected by universal newborn hearing screening program. Int J Pediatr Otorhinolaryngol.

[13] Shang Y, Hao W, Gao Z, Xu C, Ru Y, Ni D (2016). An effective compromise between cost and referral rate: a sequential hearing screening protocol using TEOAEs and AABRs for healthy newborns. Int J Pediatr Otorhinolaryngol.

[14] Gulati A, Sakthivel P, Singh I, Ramji S (2022). The hearing status of preterm infant’s ≤ 34 weeks as revealed by otoacoustic emissions (OAE) screening and diagnostic brainstem evoked response audiometry (BERA): a tertiary center experience. Indian J Otolaryngol Head Neck Surg.

[15] Hardani AK, Goodarzi E, Delphi M, Badfar G (2020). Prevalence and risk factors for hearing loss in neonates admitted to the neonatal intensive care unit: a hospital study. Cureus.

[16] Van Riper LA, Kileny PR (1999). ABR hearing screening for high-risk infants. Am J Otol.

[17] Farhat A, Ghasemi MM, Akhondian J (2015). Comparative study of hearing impairment among healthy and intensive care unit neonates in mashhad, north east iran. Iran J Otorhinolaryngol.

[18] Vashistha I, Aseri Y, Singh BK, Verma PC (2016). Prevalence of hearing impairment in high risk infants. Indian J Otolaryngol Head Neck Surg.

[19] Siddique AK, Melkundi RS, Karuppannan A, Patil S, Sreedevi N (2023). Prevalence of hearing impairment in high-risk neonates at Kalaburagi region of northern Karnataka: a hospital-based cross-sectional study. Indian J Otolaryngol Head Neck Surg.

[20] Ari-Even Roth D, Hildesheimer M, Maayan-Metzger A, Muchnik C, Hamburger A, Mazkeret R, Kuint J (2006). Low prevalence of hearing impairment among very low birthweight infants as detected by universal neonatal hearing screening. Arch Dis Child Fetal Neonatal Ed.

[21] Andrade GM, Resende LM, Goulart EM, Siqueira AL, Vitor RW, Januario JN (2008). Hearing loss in congenital toxoplasmosis detected by newborn screening. Braz J Otorhinolaryngol.

[22] Ferreira L, Sanfins MD, Pinto JD, Skarzynski PH, Skarżyńska MB, Vieira Biaggio EP (2023). Congenital toxoplasmosis and auditory disorders: a literature review. Front Psychol.

[23] Poonual W, Navacharoen N, Kangsanarak J, Namwongprom S (2016). Risk factors for hearing loss in infants under universal hearing screening program in Northern Thailand. J Multidiscip Healthc.

[24] Gouri ZU, Sharma D, Berwal PK, Pandita A, Pawar S (2015). Hearing impairment and its risk factors by newborn screening in north-western India. Matern Health Neonatol Perinatol.

[25] Keihanidost Z, Tabrizi A, Amini E, Sedaghat M, Ghahremani A, Shariat M, Kavyani Z (2018). Risk factors for hearing loss and its prevalence in neonates older than 6 months with history of hospitalization in intensive care unit. Iran J Child Neurol.

[26] Meyer C, Witte J, Hildmann A (1999). Neonatal screening for hearing disorders in infants at risk: incidence, risk factors, and follow-up. Pediatrics.

[27] Zou KH, O'Malley AJ, Mauri L (2007). Receiver-operating characteristic analysis for evaluating diagnostic tests and predictive models. Circulation.

